# Relationships between oral function, dietary intake and nutritional status in older adults aged 75 years and above: a cross-sectional study

**DOI:** 10.1186/s12889-024-18906-y

**Published:** 2024-05-31

**Authors:** Xiaoqing Wu, Yanqiu Xu, Yajun Liu, Aiguo Ma, Feng Zhong, Tianlin Gao, Jing Cai, Yong Chen, Yali Wang, Wenkai Zhou, Yan Ma

**Affiliations:** 1 Department of Nutrition and Food Hygiene, School of Public Health, Qingdao University, Qingdao, Shandong China; 2 Institute of Nutrition and Health, School of Public Health, Qingdao University, Qingdao, Shandong China; 3 School of Food Science and Biotechnology, Food Oral Processing Laboratory, Zhejiang Gongshang University, Hangzhou, Zhejiang China

**Keywords:** Older adults, Nutritional status, Dietary intake, Oral function

## Abstract

**Background:**

Malnutrition is related to impaired oral health and function that causes poor dietary intake, declining the general health of older adults. The role of dietary intake in the association between oral function and nutritional status of Chinese older adults (aged 75 and above) was examined in this cross-sectional study.

**Methods:**

Through the randomized cluster sampling method, 267 older adults living in rural areas of Qingdao, Shandong (aged 81.4 ± 4.3, 75–94 years) were chosen as the primary research participants. A Mini Nutritional Assessment - Short Form was used to determine nutritional status, and Food Frequency Questionnaire and 24-hour Food Intake Recall were used to assess dietary intake. The oral function was evaluated by analyzing the teeth, oral problems, bite force, tongue pressure, lip sealing pressure, chewing function questionnaire, whole saliva flow rate, 10-Item Eating Assessment Tool, and water swallow test.

**Results:**

Based on the MNA-SF score, it was divided into a well-nourished group and a malnutrition group, with the malnutrition group comprising 40.6% of participants. The participants in the malnutrition group showed a higher rate of xerostomia, lower bite force, tongue pressure, and lip sealing pressure, and higher Chewing Function Questionnaire and 10-Item Eating Assessment Tool scores. Furthermore, their plant fat, iron, cereals and potatoes, vegetables, fruits, and seafood intake were relatively low. The regression model indicated that exercise frequency, stroke, chewing and swallowing function, intake of vegetables and fruits were risk factors for nutritional status of older adults.

**Conclusion:**

Malnutrition was relatively common among the Chinese older adults aged 75 and above, and it was significantly correlated with exercise frequency, stroke, chewing and swallowing function, and intake of vegetables and fruits. Therefore, nutrition management should be carried out under the understanding and guidance of the oral function and dietary intake of the older adults.

**Supplementary Information:**

The online version contains supplementary material available at 10.1186/s12889-024-18906-y.

## Introduction

The world’s aging process is constantly accelerating. There were roughly 700 million older adults globally in 2019; it is predicted that this number will double by 2050 and that the proportion of the 80 + population will triple by that time [1]. Maintaining the health of older adults, particularly those over 75 years old, is crucial in our aging society. The problems with insufficient and imbalanced nutrition at this age stage, which are linked to higher mortality, greater susceptibility to infection, and worse quality of life [2], are quite concerning. Studies have reported that 12.6% of the Chinese older adults were malnourished [3], and have reported that malnutrition afflicted up to 53.0% of older adults over the age of 80 [4]. As the aging process speeds up in modern society, reducing the risk of malnutrition has emerged as a significant public health concern.

A number of reasons can lead to malnutrition in older individuals. In addition to the determinants of disease, physiological, psychological, and socioeconomic [5], previous research has also shown a close relationship between dietary intake and nutritional status in the older adults [6]. Energy and protein consumption are directly linked to malnutrition. Previous studies have shown that an imbalance in the proportion of energy consumed by carbohydrates and fats can lead to changes in body weight [7]. In addition, in the older adults, adequate intake of fruits and vegetables is associated with preventing malnutrition [8] and various diseases. Oral function is an important factor affecting dietary intake [9, 10], so to some extent, the oral cavity can also serve as a useful indicator reflecting nutrition and general health [11]. The oral and overall health status deteriorates with age and sickness [12, 13], leading to an increase in the number of difficult-to-chew food clumps over time, and ultimately changing dietary preferences and eating habits [12, 14]. The older adults tend to choose foods that are soft and easy to chew, while avoiding foods that are hard textures and rich in fiber [14, 15], which increases the intake of fat, other carbohydrates, and processed foods, and reduces the consumption of raw fruits, vegetables, nuts, and meat [15–17]. Consistently making unbalanced food choices can lead to an unhealthy diet that is high in carbohydrates and calories but low in protein, minerals, and fiber [17], which may even cause malnutrition in older adults and increase their risk of developing certain diseases, becoming feeble, or even dying [9]. Therefore, there is a relationship between oral health, function, and malnutrition in the older adults. Potential causes for this connection include the effect of inadequate nutritional intake brought on by missing dentition, insufficient saliva secretion, and chewing and swallowing issues [18–20]. All of these changes may affect the speed and effectiveness of food clumping and movement in the mouth, making older adults more vulnerable to dietary restrictions, leading to malnutrition. Because many oral health issues can be treated and dietary habits can be changed, identifying the risk factors for malnutrition is crucial to devise prevention strategies.

It is widely established that there are well-established connections between oral capability and nutritional status [21–25]. The impact of single and/or multiple oral health and function on nutritional status or the impact on food intake, however, was the only focus of several earlier studies, which gave little consideration to the role of dietary intake in nutrition. It is yet unclear what part dietary intake plays in the connection between nutritional status and oral condition. Moreover, there are currently few relevant studies paying attention to the association between oral function and nutritional status of the Chinese older adults aged 75 and above. In China, there is a lack of understanding of the severe prevalence of malnutrition among the older adults of this age stage. It is socially significant to investigate the connection between oral function and malnutrition in the Chinese older adults. From a dental standpoint, this information will prevent malnutrition and give us a scientific foundation for addressing the demands of population aging.

Insufficient dietary intake could result from impaired oral function, according to the hypothesis of this study, which could then compromise nutrition status. Therefore, this cross-sectional study’s goal was to focus on the dietary intake of the Chinese older adults aged 75 and above and to clarify its role in the association between oral condition and nutritional status.

## Methods

### Study participants

Between August 2022 and August 2023, a survey study was carried out in the Qingdao City, Shandong Province, China. The participants were selected from older adults living in rural areas who were 75 years of age or older and taking advantage of basic public health services for health management. Each participant was able to stand, eat, drink, and converse independently. Participants excluded from the survey include those whose muscles of mastication were unable to exert force and/or whose salivary glands were affected by drugs. The Qingdao University Medical College Ethics Committee granted approval for this study (Approval number: QDU-HEC-2023190). Informed consent was obtained from all participants.

**Fig. 1:**
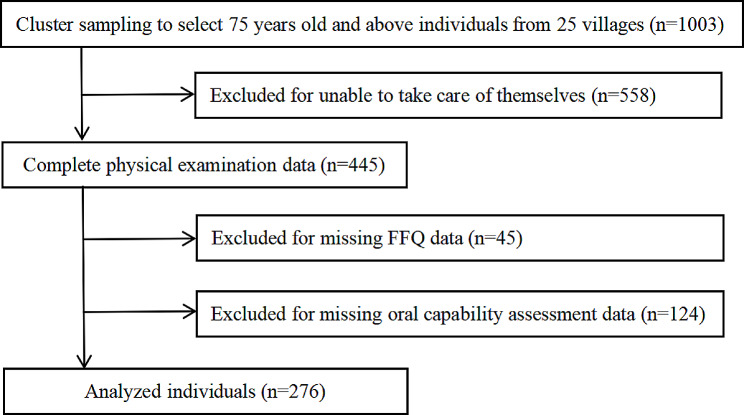
Flow chart for inclusion and exclusion

During the investigation period, 25 villages with a total of 1003 older adults over 75 years old were chosen using the randomized cluster sampling approach. We first excluded 558 persons who were unable to take care of themselves. Thus, 445 participants with complete physical examination data were enrolled, while 45 participants lacking Food Frequency Questionnaire (FFQ) data and 124 missing oral capacity assessment data were omitted. As a result, we included 276 participants in this study who were 75 years of age or older (mean age: 81.4 ± 4.3 years, 121 men and 155 women) (Fig. 1).

### Basic information

In order to gather fundamental data about participants’ sociodemographic traits and behaviors, such as gender, age, marital status, smoking history, and drinking habits, participants were asked to complete a survey questionnaire. The research investigators received standardized training before the questionnaires were distributed. Researchers obtained basic information and questionnaires from participants through face-to-face interviews.

The medical history data of chronic diseases such as hypertension and diabetes, as well as the data related to blood pressure, blood biochemistry, and other physical examination data were all gathered from a report provided by the National Basic Public Health Services database.

### Nutrition status assessment methods

A nutritional screening tool known as the Mini Nutritional Assessment - Short Form (MNA-SF) [26] is used to detect older adults who are malnourished or at risk of malnutrition. It consists of six items and the total score is 14 points, and higher scores are suggestive of normal nutritional status. Malnutrition (0–7 points), at risk of malnutrition (8–11 points), and well-nourished (12–14 points) were the three categories used to categorize nutritional status.

Given that the proportion of malnourished people in the included studies was relatively low, nutritional status was divided into a malnutrition group (at risk of malnutrition and malnutrition) and a well-nourished group (well-nourished) for appropriate statistical analysis [25, 27].

### Dietary survey

Two questionnaires were used to assess individuals’ nutritional and food group intake: FFQ and the 24-hour Food Intake Recall (24HR) [18, 28]. The FFQ used in this study is a questionnaire containing 97 questions, which has been validated and recognized in previous studies [29, 30]. The frequency and portion size of food consumption in the past three months have also been questioned. We included 9 food groups in the final analysis to preserve the study’s biggest size, including cereals and potatoes, soybean and its products, vegetables, fruits, meat, fish and seafood, eggs, fats, and oils. The rationality of selecting the main food group takes into account the research assumptions and dietary habits. Likewise, the 24HR is a similarly proven survey tool and a method of open-ended daily meal entry [31].

### Oral function assessment

The trained researcher conducted an oral function assessment and determined the number of teeth, denture use, and oral problems (xerostomia / toothache / halitosis / bleeding gums, etc.).

The Chewing Function Questionnaire (CFQ) [32] evaluated the direct or subjective effects of reduced chewing function. It reflected participants’ subjective perceived chewing function values and showed good psychometric properties [33]. It consists of ten items, with a total score of 40, and the higher the score, the greater the difficulty in chewing.

The salivary flow rate test was conducted from 9:30 a.m. to 11:30 a.m. on the test day in order to avoid circadian influence. Unstimulated (UWS) and stimulated (SWS) whole saliva were the two different types of whole saliva that were collected. When collecting UWS, participants were requested to naturally spit saliva into a sterile, disposable preservation tube via a saliva collector for 5 min. After 10 min, in order to mechanically stimulate saliva secretion, they were instructed to chew on a piece of safe, non-toxic, 5.0 × 5.0 cm Parafilm (PM-996, Bemis Inc, Neenah, USA), and then SWS was collected in the same way as UWS. Assuming a specific gravity of 1.0, the volume of the saliva sample was calculated gravimetrically after collection [34].

The bite force device is a modified laboratory-made force sensor (FlexiForce A201-100, Tekscan, South Boston, Massachusetts, USA) [35] used to measure bite force. The instrument for recording the data was a multi-meter (UT181A, UNI-T, Guangdong, China). The participants were instructed to bite on the nearest teeth based on the position of the missing first molar when one of the first molars was gone [36]. Participants who wore dentures were requested to wear them throughout the measures. Participants were instructed to exert maximal bite down on the sensor for 3 s while taking the test.

Utilizing the Iowa Oral Performance Instrument (IOPI®, Medical LLC, Redmond, Washington, USA) [37], tongue pressure (TP) and lip sealing pressure measurements were taken. IOPI consists of a tiny air-filled bulb, which is a portable pressure bulb device. When measuring TP, participants were instructed to place the bulb in the middle of the oral cavity between the tongue and the hard palate and to press as hard as they could. The bulb was then placed between their lips, and they were instructed to push their lips as hard as possible to measure lip sealing pressure. The highest pressure was recorded in kPa.

The 30mL Water Swallow Test (WST) [38], a commonly used dysphagia test, and the validated Hebrew version of 10-Item Eating Assessment Tool (EAT-10) [39] were used to screen for dysphagia. The total score of EAT-10 is 40 and higher score, which are suggestive of the more serious swallowing problem. The dysphagia threshold for EAT-10 is 3 points. According to this threshold, the subjects were categorized as dysphagic or normal. The 30 mL WST required participants to continuously consume 30 mL of water. Any coughing or clearing of the throat was a sign of dysphagia. The results were divided into five grades, with grade one being negative and grades two to five being positive.

### Statistical analysis

This study used Excel spreadsheets for data entry and used SPSS 26.0 statistical software for data management and processing. Continuous variables were shown as means and standard deviation; while counting variables were represented by percentages (%). The Kolmogorov-Smirnov test was used to determine the normality of the data distribution; the results showed that the data of this study were non-normally distributed (*p* > 0.05). Mann-Whitney and Chi-square tests were used to compare characteristics, oral function, and dietary intake between subjects with varying nutritional status. We looked at the link between oral function and dietary intake using Spearman’s rank correlation analysis. With stepwise approaches (input 0.05, remove 0.10), we ran a multiple regression analysis to ascertain the association between relevant factors and nutritional status. The score of the MNA-SF was used as the dependent variable. Select basic characteristics, oral function, and dietary intake that are closely related to nutritional status through Mann-Whitney or chi-square test as independent variables. A *P* value lower than 0.05 indicated statistical significance.

## Results

### Characteristics of subjects according to nutrition status

According to nutritional status, Table 1 displayed the characteristics of the study subjects. The older adults polled for this study had an average age of 81.4 ± 4.3 years, a mean BMI of 23.9 ± 3.9 Kg/m^2^, and 56.2% were female and 43.8% were male. In terms of nutritional status, the MNA-SF identified 6 (2.2%), 106 (38.4%), and 164 (59.4%) individuals as malnutrition, at risk of malnutrition, and well-nourished, respectively. Thus, the malnutrition group consisted of 112 (40.6%) participants. In comparison to the well-nourished group, the malnutrition group revealed associated with stroke (*p* = 0.005), older age (*p* = 0.004), lower BMI (*p* < 0.001), less exercise (*p* < 0.001), lower hemoglobin (*p* = 0.043), lower triglyceride (*p* < 0.001), and higher HDL (*p* < 0.001). Between the groups, there were no discernible relationships for the other factors.

**Table 1:** Characteristics of subjects according to nutrition status

Variables	Total (*n* = 276)	Well-nourished group (*n* = 164)	Malnutrition group (*n* = 112)	*p*-Value
Age (years)*	81.4 ± 4.3	80.8 ± 4.0	82.3 ± 4.5	0.004
BMI (Kg/m^2^)*	23.9 ± 3.9	25.5 ± 3.3	21.6 ± 3.6	0.000
Gender				0.335
Female	155 (56.2)	96 (58.5)	59 (52.7)	
Male	121 (43.8)	68 (41.5)	53 (47.3)	
Marital status				0.662
Married	141 (51.1)	82 (50.0)	59 (52.7)	
Widowed and others	135 (48.9)	82 (50.0)	53 (54.8)	
Educational level				0.560
Illiterate	142 (51.4)	82 (50.0)	60 (53.6)	
Primary school or above	134 (48.6)	82 (50.0)	52 (46.4)	
Living arrangement				0.507
Alone	115 (41.7)	71 (43.3)	44 (39.3)	
With Family	161 (58.3)	93 (56.7)	68 (60.7)	
Exercise Frequency (times/week)*	2.8 ± 2.7	3.2 ± 2.7	2.2 ± 2.6	0.000
Smoking experience				0.200
Yes	89 (32.2)	48 (29.3)	41 (36.6)	
No	187 (67.8)	116 (70.7)	71 (63.4)	
Drinking experience				0.340
Yes	92 (33.3)	51 (31.1)	41 (36.6)	
No	184 (66.7)	113 (68.9)	71 (63.4)	
Blood Pressure				
Systole (mmHg)	146.2 ± 17.2	147.7 ± 17.2	143.9 ± 17.0	0.094
Diastole (mmHg)	81.3 ± 10.2	82.0 ± 10.6	80.3 ± 9.6	0.142
Pulse Pressure (mmHg)	64.9 ± 14.7	65.7 ± 15.5	63.6 ± 13.5	0.255
Blood biochemical indicators				
Hemoglobin*	134.5 ± 13.3	135.9 ± 13.0	132.5 ± 13.6	0.043
Total cholesterol	5.6 ± 1.1	5.6 ± 1.1	5.6 ± 1.1	0.930
Triglyceride*	1.2 ± 0.7	1.4 ± 0.7	1.0 ± 0.4	0.000
LDL	2.9 ± 0.7	3.0 ± 0.7	2.9 ± 0.8	0.474
HDL*	1.6 ± 0.4	1.6 ± 0.4	1.8 ± 0.4	0.000
Number of chronic diseases				0.926
None	73 (26.4)	42 (25.6)	31 (27.7)	
Single	141 (51.1)	85 (51.8)	56 (50.0)	
Multiple	62 (22.5)	37 (22.6)	25 (22.3)	
Number of drugs				0.290
None	101 (36.6)	60 (36.6)	41 (36.6)	
Single	89 (32.2)	58 (35.4)	31 (27.7)	
Multiple	86 (31.2)	46 (28.0)	40 (35.7)	
Comorbidity Disease				
Hypertension	176 (63.8)	107 (65.2)	69 (61.6)	0.537
Diabetes mellitus	49 (17.8)	34 (20.7)	15 (13.4)	0.117
Cardiovascular disease	36 (13.0)	21 (12.8)	15 (13.4)	0.887
Stroke**	12 (4.3)	2 (1.2)	10 (8.9)	0.005

The number of participants (%) or the mean *±* standard deviation was used to present the data. *: The Mann-Whitney test revealed significant differences; **: the chi-square test revealed significant disparities. BMI: Body Mass Index; LDL: Low-Density Lipoprotein; HDL: High-Density Lipoprotein; Exercise frequency: the frequency of exercise every week; Smoking experience: Participants who smoked or had a smoking history; Drinking experience: Participants who drank or had a drinking alcohol history

### The association between oral function and nutritional status

The parameters of oral function characteristics and their relationship to nutritional status were displayed in Table 2. In terms of oral health issues, xerostomia in the previous 6 months had the highest prevalence (46.0%) and was more frequently reported by malnourished individuals than by those who were not (*p* = 0.037). While the malnutrition group had a considerably higher CFQ score (*p* < 0.001) than the well-nourished group, the mean average of the bite force (*p* < 0.001), TP (*p* = 0.003), and lip sealing pressure (*p* = 0.001) among participants in the malnutrition group was significantly lower than that of the well-nourished group. For dysphagia screening, according to EAT-10 results, the older adults who were malnourished had a higher likelihood of having dysphagia (*p* < 0.001). The nutritional status was not correlated with other parameters.

**Table 2:** The association between oral function and nutritional status

Variables	Total (*n* = 276)	Well-nourished group (*n* = 164)	Malnutrition group (*n* = 112)	*p*-Value
**Number of teeth**				0.118
Both jaws > 7 teeth	111 (40.2)	74 (45.1)	37 (33.0)	
At least one jaw with 1–7 teeth	118 (42.8)	63 (38.4)	55 (49.1)	
No teeth	47 (17.0)	27 (16.5)	20 (17.9)	
**Dentures**				0.081
No dentures	127 (46.0)	66 (40.2)	61 (54.5)	
Partial dentures	80 (29.0)	54 (32.9)	26 (23.2)	
Complete dentures	44 (15.9)	26 (15.9)	18 (16.1)	
Combined dentures	25 (9.1)	18 (11.0)	7 (6.3)	
**Oral problems**				
Xerostomia**	127 (46.0)	67 (40.9)	60 (53.6)	0.037
Toothache	69 (25.0)	45 (27.4)	24 (21.4)	0.257
Halitosis	44 (15.9)	26 (15.9)	18 (16.1)	0.961
Bleeding gums	35 (12.7)	21 (12.8)	14 (12.5)	0.940
Blisters or sores in the mouth	39 (14.1)	22 (13.4)	17 (15.2)	0.680
Lost, loose, broken teeth	69 (25.0)	46 (28.0)	23 (20.5)	0.157
Dental caries	111 (40.2)	63 (38.4)	48 (42.9)	0.460
**Oral function**				
Bite force (N)*	86.3 ± 99.0	102.7 ± 108.6	62.3 ± 77.4	0.000
Tongue pressure (kPa)*	19.7 ± 12.0	21.4 ± 11.6	17.3 ± 12.3	0.003
Lip sealing pressure (kPa)*	17.0 ± 8.0	18.5 ± 8.3	14.9 ± 7.0	0.001
Score of CFQ*	10.3 ± 5.9	8.9 ± 5.3	12.4 ± 6.1	0.000
FR-UWS (ml/min)	0.4 ± 0.3	0.5 ± 0.3	0.4 ± 0.3	0.871
FR-SWS (ml/min)	0.9 ± 0.5	1.0 ± 0.6	0.9 ± 0.5	0.267
Grade of EAT-10**				0.000
< 3	212 (76.8)	141 (86.0)	71 (63.4)	
≥ 3	64 (23.2)	23 (14.0)	41 (36.6)	
Grade of WST				0.062
1	185 (76.0)	118 (72.0)	67 (59.8)	
2	80 (29.0)	42 (25.6)	38 (33.9)	
3	11 (4.0)	4 (2.4)	7 (6.3)	

The number of participants (%) or the mean *±* standard deviation was used to present the data. *: The Mann-Whitney test revealed significant differences; **: the chi-square test revealed significant disparities. CFQ: Chewing Function Questionnaire; FR-UWS: unstimulated whole saliva flow rate; FR-SWS: stimulated whole saliva flow rate; EAT-10: 10-Item Eating Assessment Tool; WST: Water Swallow Test

### The association between dietary intake and nutritional status

Comparing the nutrient and food intake between the two groups was shown in Table 3. In comparison to the well-nourished group, the malnourished group’s intake of plant fat (*p* = 0.046) and iron (*p* = 0.025) was much lower. However, there was no significant difference in various vitamins. Furthermore, the malnutrition group had significantly lower food intake, such as cereals and potatoes, vegetables, fruits, fish, and seafood. In addition, it was discovered that the vegetable intake of older adults with dysphagia in the malnutrition group was lower among the two groups, while the intake of vegetables, fruits, and fish and seafood of older adults with normal swallowing function in the well-nourished group was higher among the two groups (see Additional file 1).

**Table 3:** The association between dietary intake and nutritional status

Nutrient and Food Groups	Total (*n* = 276)	Well-nourished group (*n* = 164)	Malnutrition group (*n* = 112)	*p*-Value
**Nutrient Intake**				
Energy (kcal/d)	1307.4 ± 548.9	1348.6 ± 592.2	1247.0 ± 474.6	0.315
Carbohydrates (g/d)	168.2 ± 63.7	171.5 ± 65.7	163.3 ± 60.6	0.466
Dietary Fiber (g/d)	7.9 ± 4.5	8.4 ± 5.1	7.1 ± 3.2	0.050
Protein Total (g/d)	48.7 ± 20.1	50.0 ± 20.9	46.8 ± 18.6	0.240
Animal Protein	20.2 ± 13.9	20.5 ± 14.4	19.8 ± 13.0	0.886
Plant Protein	28.5 ± 14.1	29.5 ± 14.7	27.0 ± 13.1	0.264
Fat Total (g/d)	46.5 ± 33.3	49.2 ± 36.9	42.5 ± 26.7	0.226
Animal Fat	31.4 ± 26.1	32.6 ± 29.9	29.6 ± 19.2	0.871
Plant Fat*	15.1 ± 22.6	16.6 ± 23.1	13.0 ± 21.9	0.046
Sodium (mg/d)	887.4 ± 722.7	899.2 ± 752.3	870.0 ± 680.1	0.969
Potassium (mg/d)	1217.8 ± 513.3	1261.2 ± 567.4	1154.3 ± 416.0	0.241
Calcium (mg/d)	343.3 ± 191.3	352.5 ± 194.0	329.7 ± 187.3	0.320
Magnesium (mg/d)	246.4 ± 131.1	257.4 ± 135.6	230.2 ± 123.0	0.072
Iron (mg/d)*	12.5 ± 9.5	13.3 ± 10.4	11.4 ± 8.0	0.025
Manganese (mg/d)	4.5 ± 2.6	4.8 ± 3.1	4.1 ± 1.8	0.126
Zinc (mg/d)	6.6 ± 3.5	6.8 ± 3.3	6.5 ± 3.8	0.295
Copper (mg/d)	1.2 ± 1.5	1.2 ± 1.4	1.1 ± 1.7	0.358
Phosphorous (mg/d)	767.8 ± 320.6	785.4 ± 334.0	742.1 ± 299.6	0.268
Selenium (µg/d)	45.8 ± 28.1	45.9 ± 25.3	45.6 ± 32.0	0.610
Retinol (µgRAE/d)	393.3 ± 360.0	403.2 ± 381.9	378.7 ± 326.3	0.992
Vit. B1 (mg/d)	0.5 ± 0.3	0.5 ± 0.3	0.4 ± 0.2	0.193
Vit. B2 (mg/d)	0.6 ± 0.3	0.6 ± 0.3	0.6 ± 0.3	0.599
Niacin (mgNE/d)	10.7 ± 6.4	11.0 ± 6.7	10.1 ± 5.9	0.449
Vit. C (mg/d)	72.4 ± 61.0	74.3 ± 70.9	69.8 ± 42.7	0.787
Vit. E (mg α-TE/d)	11.2 ± 11.4	12.0 ± 11.7	10.1 ± 11.0	0.115
**Food Groups (g/day)**				
Cereals and Potatoes*	239.8 ± 93.2	251.0 ± 98.3	223.4 ± 82.9	0.015
Soybean and its products	20.9 ± 34.1	22.1 ± 34.6	19.2 ± 33.4	0.424
Vegetables*	227.8 ± 95.9	243.6 ± 96.4	204.7 ± 90.7	0.001
Fruits*	85.0 ± 87.3	99.3 ± 95.0	64.1 ± 69.7	0.000
Meat	59.9 ± 54.3	64.6 ± 58.4	52.9 ± 47.2	0.151
Fish and Seafood*	21.3 ± 31.0	24.0 ± 32.0	17.3 ± 29.2	0.020
Milk products	102.7 ± 115.1	107.6 ± 115.5	95.4 ± 114.8	0.470
Eggs	38.2 ± 22.4	38.7 ± 21.2	37.4 ± 24.2	0.162
Fats and oils	24.2 ± 6.1	24.7 ± 6.3	23.6 ± 5.6	0.167

The mean *±* standard deviation is used to present the data. *: The Mann-Whitney test revealed significant differences. Vit.: Vitamin

### The correlation between dietary intake and oral function

Even though there were significant differences between the two groups (as shown in Table 3), the relationship between oral function and food item intake was still unclear. The results of Table 4 showed that the bite force and vegetable intake of participants performed a positive association (*P* = 0.008), the grade of EAT-10 and plant fat intake (*P* = 0.028), and iron (*P* = 0.042) intake exhibited a negative correlation. The score of CFQ exhibited a negative correlation with plant fat (*P* = 0.005), iron (*P* = 0.012), cereals and potatoes (*P* = 0.019), vegetables (*P* < 0.001), fruits (*P* = 0.001), fish and seafood (*P* = 0.025). Therefore, the score of CFQ was the only factor significantly correlated with all of these parameters of dietary intake.

**Table 4:** The correlation between dietary intake and oral function

Variables	Bite force		TP		Lip sealing pressure		Score of CFQ		Grade of EAT-10	
	r	*p*	r	*P*	r	*p*	r	*p*	r	*p*
plant fat	0.045	0.461	-0.047	0.433	0.074	0.218	-0.170**	0.005	-0.133*	0.028
Iron	0.028	0.649	-0.103	0.089	0.019	0.748	-0.151*	0.012	-0.123*	0.042
Cereals and Potatoes	0.093	0.125	-0.027	0.660	0.081	0.179	-0.141*	0.019	-0.077	0.200
Vegetables	0.160**	0.008	-0.090	0.134	-0.023	0.706	-0.232**	0.000	-0.067	0.269
Fruits	-0.001	0.984	0.030	0.619	0.095	0.115	-0.191*	0.001	-0.063	0.300
Fish and Seafood	0.088	0.143	0.093	0.125	0.111	0.065	-0.135*	0.025	-0.013	0.832

R: Spearman correlation coefficient, *: *p* < 0.05, **: *p* < 0.001

### Associations of oral function and dietary intake with nutritional status

According to the results of the multiple regression analysis, as shown in Table 5, the factors associated with MNA-SF score were exercise frequency (*P* = 0.001, Beta = 0.186), stroke (*P* = 0.033, Beta = -0.116), score of CFQ (*P* < 0.001, Beta = -0.228) and EAT-10 (*P* < 0.001, Beta = -0.214), and intake of vegetable (*P* = 0.047, Beta = 0.107) and fruit (*P* = 0.015, Beta = 0.130). This indicates that whereas exercise frequency, vegetables and fruits intake had a positive correlation with MNA-SF score, stroke chewing and swallowing function had a negative correlation.

**Table 5:** Influencing Factors of the Mini Nutritional Assessment score for the older adults

Variables	Nonstandard coefficient		Standardization coefficient	t	*P*	95% Confidence Interval for B
	B	Standard error	Beta			
Exercise Frequency	0.120	0.034	0.186	3.503	0.001	0.053–0.188
Stroke	-0.997	0.466	-0.116	-2.138	0.033	(-1.915)-(-0.079)
Score of CFQ	-0.068	0.017	-0.228	-4.025	0.000	(-0.101)-(-0.034)
Score of EAT-10	-0.244	0.064	-0.214	-3.780	0.000	(-0.371)-(-0.117)
Vegetables	0.002	0.001	0.107	1.997	0.047	0.000-0.004
Fruits	0.003	0.001	0.130	2.449	0.015	0.001–0.005
Constant	12.820	0.604		21.237	0.000	11.631–14.008

Multiple regression analyses with stepwise methods (input: 0.05; removal: 0.10). *R* = 0.519, R^2^ = 0.269, After adjustment R^2^ = 0.253, F = 16.496, *P* < 0.001. Durbin-Watson = 1.964. Only the variables in the equation are displayed in the table. The score of the MNA-SF was used as the dependent variable. Tables 1, 2 and 3 factors that had a significantly correlation with nutritional status were entered as independent variables

## Discussion

This study focused on dietary intake to evaluate its role in the relationship between oral function and nutritional status. The study found that chewing function was the only factor substantially connected with plant fat, iron, cereals and potatoes, vegetables, and fruits among the Chinese older adults aged 75 and above. Additionally, significant explanatory factors affecting the nutritional status included dysphagia, chewing function, frequency of exercise, stroke, and intake of vegetables and fruits. Our study hypothesizes that decreased oral function makes it difficult for older adults to chew or swallow various foods, leading to a decrease in dietary nutrient intake and resulting in malnutrition. The findings of our study suggest that maintaining good oral function is essential for enhancing nutrient intake.

The rates of those who were malnourished or at risk of malnutrition in the sample of the older adults in the current study who were 75 years of age and older were 2.2% and 38.4%, respectively. According to earlier study in Israel [40], which had a small sample size (180 participants), 17.8% of senior citizens aged 65 or older who were assessed by MNA-SF were malnourished or at risk of malnutrition. Another study conducted in China found that among the general population aged 65 and above, 36.4% of people were at risk of malnutrition [41]. These two studies both reveal lower malnutrition risk prevalence than the current analysis. The older characteristics of the participants we included may be responsible for the high rate. It is precisely because as age increases, the risk of malnutrition is higher [42].

The results of this investigate showed that participants in the malnutrition group were assumed to have worse masticatory performance and swallowing function because CFQ and EAT-10 scores were negatively correlated with MNA-SF scores. Pronunciation, swallowing, and retaining dentition all depend on lip sealing pressure and TP [43]. The bite force can reflect the number of teeth, the effect of wearing removable dentures, and the strength of facial muscles [10]. Bite force, TP, and lip sealing pressure are all indicators of masticatory performance that reflect muscular strength. Although these three factors were not significant explanatory variables affecting the nutritional status of participants in this study, there were statistical differences between the two groups. Perhaps this is the case because these three factors did not directly affect nutritional status, but rather affect nutritional status by altering chewing function. According to studies, TP improves with practice [44], and dentition repair can increase bite force. Therefore, regularly assessing oral muscle strength and striving to maintain and improve chewing ability among the older adults may enhance physical fitness. On the other hand, salivary secretion is essential for oral functions including chewing and swallowing. This study revealed a statistically significant difference in xerostomia between the two groups, with more participants in the malnutrition group experiencing xerostomia. Although xerostomia is a subjective sensation of dry mouth, it is not necessarily connected to a decreased saliva flow rate [45]. As insalivation is crucial for the decomposition of food and the formation of food clumps during eating [19, 46, 47], xerostomia that is accompanied by hyposalivation may have a detrimental effect on dietary intake. This could account for the interaction between xerostomia with the deterioration in nutritional status that we observed in our study.

Chewing and swallowing are essential functions for nutrient intake, and impaired chewing and swallowing abilities can lead to poor health, including malnutrition [20, 25, 40]. According to a number of observational studies, the low bite force has been significantly linked to a decrease in vegetables, fruits, fish, protein, dietary fiber, and most vitamins and minerals intake [10]. This study made the assumption that older adults with fewer teeth and lower bite force would unknowingly adopt a habitually unhealthy diet, such as favoring food that is easy to chew, which would change their food preferences, result in a poor diet, and even worsen their nutritional status. But in contrast to what we anticipated, our study found no connection between the number of teeth and nutritional status. It could be because older adults commonly have most of their teeth missing and have dentures fitted, and the participants in prior studies were younger than those in our study [22, 23, 48]. Therefore, the number of teeth alone cannot be used to determine how well someone can chew. For individuals with dysphagia, relevant studies have shown a significant reduction in vitamin E and magnesium intake [18], and older adults with dysphagia were found on malnutrition [49]. This was similar to the findings of our study that the plant fat, vegetables, iron, and cereals and potatoes intakes of the older adults with dysphagia in the malnutrition group were lowest among the groups. Therefore, closely monitoring the dietary intake of the older adults with chewing and/or swallowing difficulties to ensure adequate consumption may be necessary for improving overall health and enhanced quality of life.

According to our findings, the intakes of fruits and vegetables were positively correlated with nutritional status, which is consistent with the findings of Aparicio et al. [8], who found a high correlation between consuming 400 g/d of fruits and vegetables and better nutritional status. Moreover, participants in the malnutrition group consumed less fruit and vegetables than the 300–500 g/d and 200–350 g/d, respectively, recommendations in the Chinese Residents Dietary Guidelines (2022). Some studies have suggested that increased intake of fruits and vegetables has been linked to a reduced risk of cardiovascular disease [50, 51], stroke [52], and cognitive decline [53]. The current study, however, took into account that older adults chose soft, readily chewable foods that were high in calories but low in fiber, vitamins, and protein [9]. Consuming these meals over an extended period of time may cause a shortage of certain nutrients, and this nutritional deficiency could ultimately lead to an increase in the prevalence of numerous health issues, including malnutrition.

Hence, the Chinese government should implement targeted health guidance and health education, requiring all medical staff in primary hospitals or paramedics to conduct an early assessment of oral functions and dietary nutrition intake, conduct intervention and graded management for the older adults, and instruct the older adults to improve their lifestyles including the balanced dietary intake, learned to handle difficult-to-chew foods (changing food texture, extending cooking time, etc.), increased physical activities. In addition, primary healthcare offers home dental visits and regularly screens for oral health and function. At present, the Chinese government is making great efforts to improve the dietary intake of the older adults and developing specialized foods for them, such as YISHI Food. These actions will raise the understanding of the need of oral healthcare among the older adults, boost their nutritional and physical well-being, and ultimately offer support and guidance for developing foods that could satisfy the nutritional requirements of an aging diet.

Compared with early studies, this study analyzed the oral health and functions from the viewpoints of teeth, salivation, chewing, and swallowing. It was found the link between oral health and the risk of malnutrition, which could be measured by the oral function. Another significant feature was that the research focuses on the Chinese older adults aged 75 and above, which can provide more comprehensive solutions to the increasingly serious malnutrition problem in an aging society. At the same time, two restrictions on our study must also be taken into consideration. First, because the 24HR and FFQ were both self-report questionnaires with memory bias, care had to be taken when interpreting the results because they could have inflated or underestimated dietary intake. Second, there were no causal links between dietary intake, oral function, and nutritional status because of the observational design of the study. Hence, further follow-up investigations are required in the future to clarify the significance of oral function in nutrition.

## Clinical implications

Given the importance of dietary intake and oral function in the nutritional status of the older adults, it is crucial to ensure dietary balance and improve chewing and swallowing function. From the perspective of older adults, the results of this study will increase awareness of oral function and the change of their lifestyle, which can directly and/or indirectly reduce the risk of malnutrition in the older adults. From the perspective of society, this study can draw attention to the dietary safety, promote development of the food for older adults, and promote to establish dietary grade and texture standards for the older adults. It should be emphasized that this study is a cross-sectional design, which limits causal judgment and further exploration is needed in the future.

## Conclusions

The main related factors of nutritional status include exercise frequency, stroke, chewing and swallowing function, and intake of vegetables and fruits. Maintaining oral function and balanced dietary intake are crucial for reducing the risk of malnutrition. Thus, nutrition management should be carried out under the understanding and guidance of the oral function and dietary intake of the older adults. In addition, we highlight the importance of developing specialized foods that are easy to chew for the older adults, as this can ensure their dietary intake in the event of impaired oral function, thereby achieving better nutritional intervention outcomes. In China, standards of food and dietary management for the older adults have been formulated. In the future, the development and promotion of specialized foods for older adults have now become imperative.

### Electronic supplementary material

Below is the link to the electronic supplementary material.

Supplementary Material 1

## Data Availability

Due to the ongoing article writing, the datasets created during and/or analyzed during the current investigation are not yet publicly available, however they are available from the corresponding author upon justifiable request.

## Abbreviations

TP: Tongue Pressure
FFQ: Food Frequency Questionnaire
MNA-SF: Mini Nutritional Assessment- Short Form
24HR: 24-hour Food Intake Recall
CFQ: Chewing Function Questionnaire
UWS: Unstimulated Whole Saliva
SWS: Stimulated Whole Saliva
IOPI®: Iowa Oral Performance Instrument
WST: Water Swallow Test
EAT-10: 10-Item Eating Assessment Tool
BMI: Body Mass Index
LDL: Low-Density Lipoprotein
HDL: High-Density Lipoprotein
FR-UWS: Unstimulated Whole Saliva Flow Rate
FR-SWS: Stimulated Whole Saliva Flow Rate
Vit.: Vitamin
