# Supplementary material for: Relationships between oral function, dietary intake and nutritional status in older adults aged 75 years and above: a cross-sectional study
Source: BMC Public Health. 2024 May 31;24:1465. doi: 10.1186/s12889-024-18906-y (PMC11143706; doi:10.1186/s12889-024-18906-y)
Supplement: Supplementary file 1 — Supplementary Material 1 [file 12889_2024_18906_MOESM1_ESM.docx]

**Appendix**

Table A1. Comparison of nutrient and food intake among groups by swallowing function.

| Nutrient and Food Groups | Total (n=276) | Well-nourished group (n=164) | Malnutrition group (n=112) | p-Value |
| --- | --- | --- | --- | --- |
| **Normal swallowing function** |  |  |  |  |
| Age (Years) | 81.0 ± 4.0 | 80.7 ± 3.9 | 81.7 ± 4.1 | 0.072 |
| Plant Fat (g/d) | 16.6 ± 24.3 | 17.4 ± 23.7 | 15.1 ± 25.3 | 0.110 |
| Iron (mg/d) | 13.1 ± 10.5 | 13.7 ± 11.1 | 12.1 ± 9.1 | 0.134 |
| Cereals and Potatoes | 243.7 ± 92.0 | 251.9 ± 96.8 | 227.6 ± 79.9 | 0.096 |
| Vegetables* | 230.6 ± 93.2 | 239.8 ± 92.6 | 212.4 ± 92.4 | 0.029 |
| Fruits* | 88.4 ± 90.5 | 103.2 ± 99.6 | 59.2 ± 59.8 | 0.001 |
| Fish and Seafood* | 20.4 ± 25.8 | 23.4 ± 28.7 | 14.4 ± 17.2 | 0.024 |
| **Dysphagia** |  |  |  |  |
| Age (Years) | 82.7 ± 4.9 | 81.4 ± 4.5 | 83.5 ± 4.9 | 0.108 |
| Plant Fat (g/d) | 10.0 ± 15.3 | 11.5 ± 18.2 | 9.1 ± 13.5 | 0.905 |
| Iron (mg/d) | 10.4 ± 4.9 | 10.9 ± 3.9 | 10.2 ± 5.5 | 0.200 |
| Cereals and Potatoes | 225.8 ± 96.5 | 245.8 ± 109.3 | 214.5 ± 88.0 | 0.104 |
| Vegetables* | 217.7 ± 104.4 | 266.8 ± 116.4 | 190.1 ± 86.9 | 0.009 |
| Fruits | 72.9 ± 74.8 | 76.1 ± 55.8 | 71.0 ± 84.2 | 0.275 |
| Fish and Seafood | 44.6 ± 37.5 | 45.1 ± 31.3 | 44.3 ± 41.0 | 0.475 |

The mean *±* standard deviation is used to present the data. *: The Mann-Whitney test revealed significant differences.
